# Emotions, Feelings, and Experiences of Social Workers While Attending to Vulnerable Groups: A Qualitative Approach

**DOI:** 10.3390/healthcare9010087

**Published:** 2021-01-17

**Authors:** María Dolores Ruiz-Fernández, Rocío Ortiz-Amo, Elena Andina-Díaz, Isabel María Fernández-Medina, José Manuel Hernández-Padilla, Cayetano Fernández-Sola, Ángela María Ortega-Galán

**Affiliations:** 1 Department of Nursing, Physiotherapy and Medicine, University of Almeria, 04120 Almeria, Spain; mrf757@ual.es (M.D.R.-F.); 29rocii@gmail.com (R.O.-A.); j.hernandez-padilla@ual.es (J.M.H.-P.); cfernan@ual.es (C.F.-S.); 2 Department of Nursing and Physiotherapy, University of León, 24071 León, Spain; 3 SALBIS Research Group, University of León, 24071 León, Spain; 4 EYCC Research Group, University of Alicante, 03690 Alicante, Spain; 5 Adult, Child and Midwifery Department, School of Health and Education, Middlesex University, London NW4 4BT, UK; 6 Faculty of Health Sciences, University Autónoma of Chile, Temuco 3580000, Chile; 7 Department of Nursing, University of Huelva, 21007 Huelva, Spain; angelaortega96@gmail.com

**Keywords:** social worker, vulnerability, social and health care setting, qualitative research

## Abstract

Social workers in the community setting are in constant contact with the suffering experienced by the most vulnerable individual. Social interventions are complex and affect social workers’ emotional well-being. The aim of this study was to identify the emotions, feelings, and experiences social workers have while attending to individuals in situations of vulnerability and hardship. A qualitative methodology based on hermeneutic phenomenology was used. Six interviews and two focus group sessions were conducted with social workers from the community social services and health services of the Andalusian Public Health System in the province of Almería (Spain). Atlas.ti 8.0 software was used for discourse analysis. The professionals highlighted the vulnerability of certain groups, such as the elderly and minors, people with serious mental problems, and people with scarce or no economic resources. Daily contact with situations of suffering generates a variety of feelings and emotions (anger, sadness, fear, concern). Therefore, more attention should be paid to working with the emotions of social workers who are exposed to tense and threatening situations. Peer support, talking, and discussions of experiences are pointed out as relevant by all social workers. Receiving training and support (in formal settings) in order to learn how to deal with vulnerable groups could be positive for their work and their professional and personal quality of life.

## 1. Introduction

According to the World Health Organization (WHO), vulnerability is the degree to which a population, individual, or organization is unable to anticipate, cope with, resist, and recover from the impacts of disasters [1]. The concept of vulnerability refers to those sectors or groups of the population that, due to their age, sex, marital status, or ethnic origin, are in a risky condition that prevents them from accessing development and better welfare conditions [2]. These people are suffering or undergoing a painful experience [3] and turn to social services for a solution [4,5]. The care they receive may come from community social services [6] or, more specifically, from integrated social services in the health field [7].

Social workers are the frontline professionals of social services [8], that is, the first professionals in charge of meeting the demands of users upon arrival at care services [9]. They therefore play a fundamental role in the care trajectory of individuals in situations of need and social vulnerability [10].

The number of service users with very complex demands is quite high [11]. As a result, in daily practice, social work professionals find themselves in constant contact with individuals who are experiencing considerable social challenges [12,13]. Professionals are confronted with the task of promoting equality and well-being for individuals [14]. However, the interventions carried out with users are based on the traditional social intervention model of social services [15]. This strategy provides material and/or financial resources to users, which could help them to escape from that particular situation [16]. In this case, this means that the actual implementation of this model would be carried out to a greater or lesser extent, depending on the resources available to the state in question [17].

Professionals witness the suffering and despair of those most in need [18,19]. This situation triggers professionals’ emotions and feelings, the first being understood as the automatic and uncontrollable response to a stimulus and the feelings being the conscious evaluation of the emotion or experience suffered by the individual [20]. It is quite common that the demands made are greater than the resources available to manage them or that they need to be met faster than is possible due to the administrative procedures involved [21]. In addition, the vulnerability of some groups must be added to this context [22]. According to WHO, children, the elderly, and people who are ill are particularly vulnerable. Poverty is a major contributor to vulnerability [23]. In this sense, different studies establish that minors and the elderly are among the most vulnerable populations [24]. Thus, the occupational and professional commitment to these groups of individuals becomes even greater [25].

These repeated working conditions, alongside the contact with the person’s suffering, have repercussions on the professionals’ well-being [26,27,28], causing stress, emotional discomfort, and even vicarious trauma, defined as “those emotions and behaviours resulting from the interaction with traumatic events experienced by others” [29,30]. This is known as the cost of the emotional impact of caring [31,32], that is, the price professionals pay in the process of helping people in situations of intense suffering or trauma. According to the stress process models, some of the resources that potentially serve as a protective barrier include social support, the repertoire of confrontations, and some self-concepts such as self-esteem [33,34,35]. Other protective resources with the ability to significantly reduce the harmful consequences of existing stressors are the mastery and control of existing circumstances and mutual support among the professionals themselves [36,37].

According to the reviewed literature, research on social work has traditionally focused on professional performance and how this performance affects intervention subjects. However, there are fewer studies on the impact that the suffering of users has on social workers [38,39,40,41]. In recent years, there has been an increase in research, from a quantitative paradigm, in order to describe the working conditions of this group of professionals and their professional needs [42,43,44]. Despite this, research from a qualitative paradigm, from the perspective of social workers themselves, is still scarce. In particular, it reflects the emotional situation they experience in the development of their work, and seeks to delve into the experiences, the consequences, and what they would need, in order to continue helping in a sustainable way [45,46,47].

Bearing this in mind, the main objective of this research was to identify the social workers’ emotions, feelings, and experiences while attending to individuals in situations of vulnerability and hardship. Specifically, two secondary objectives were raised: to learn about the situations causing discomfort and suffering, as well as about the consequences of the same on the social workers themselves, and to inquire about the needs and resources of professionals so as to meet the demands of their work.

## 2. Materials and Methods

### 2.1. Approach

In the present study, a qualitative design based on a phenomenological–hermeneutic approach was used. According to Van Manen [48], this approach allows the study of non-conceptualized experiences lived by people, as well as the meaning of these experiences. Thus, it was possible to perform an in-depth analysis of the daily work experiences of social workers in community social services and health services. Their feelings and perceptions about the implementation of social interventions involving vulnerable groups of individuals were explored and interpreted.

### 2.2. Recruitment and Sampling

Participants in the study were the social workers at the community social services and health services of the Andalusian Public Health System in the province of Almería (Spain). Community social services in Andalusia are distributed as follows: In the capitals and cities of more than 20,000 inhabitants, the municipalities carry out the management of these services. In cities with less than 20,000 inhabitants, the provincial council carries out the management. Specifically, in Almeria city, there are 4 community social services centers managed by the city council. Regarding the Andalusian Health Service, in the capital city of Almería, there are 13 health centers (6 of these are centers with a full-time social worker). We selected social workers employed at three community social services centers in Almeria and social workers employed at the six health centers.

A total of 20 social workers participated. Of these, 11 worked in community social services centers and 9 worked in the Andalusian Health Service.

The inclusion criteria were the following: holding a stable position as a social work professional in community social services and/or the Andalusian Health Service, having a professional career or experience of no less than eight years, and regularly providing assistance to individuals in need of social services. The following professionals were excluded: professionals with temporary employment contracts or little work experience (less than eight years), professionals in managerial positions, professionals who had no contact with people in vulnerable situations, and professionals who had any psychological impairment that made it difficult for them to provide information.

Convenience sampling was the sampling method used. To recruit as many participants as possible, a snowball sampling procedure was used: a professional was contacted, who, in turn, would contact other professionals willing to participate [49]. First, the director of an urban community social services center was contacted by telephone. The study was explained to her, and a brief summary of the study, along with authorization from the Ethics and Research Commission, was sent to her via email. Subsequently, the director of the center informed her colleagues of the study and invited them to participate. Finally, the director contacted other directors of other centers, who then followed the same procedure.

The social workers at the health centers were contacted by a mental health social worker and a case manager nurse working at an urban health center. Both professionals were responsible for providing the health and social care workers with information about the study. Once they agreed to participate in the research, the participants were contacted to arrange a meeting.

When selecting participants, gender diversity was sought, although there were few male social workers among the centers’ staff. In Spain, the social work profession is predominantly female, so the sample (a larger number of women) could be considered representative. Equal representation of health and community social workers was also sought.

### 2.3. Data Collection

In-depth interviews and focus groups were the information-gathering techniques used. Two focus group sessions (with a total of seven people in each group) and six in-depth interviews were carried out. The two focus groups were conducted by a researcher and a collaborator, who had received specific training by specialists. The discourses were taped for transcription.

First, two focus group sessions were held in February 2019. One focus group session was held in the meeting room of an urban community social services center, and the other focus group was held in the multipurpose room of a social services center in the province of Almería (Spain). The groups comprised professionals working in both services (community and health services) to ensure that professionals from both sectors were included in each focus group. The researcher led the group, while the collaborator wrote down in a field notebook those observations that could be useful in subsequent analysis. The session began with an exercise that prompted discussion and dialogue among the members of the group: “Describe your day-to-day work experiences with individuals in vulnerable situations.” Finally, the conclusions were summarized, and the members were thanked for their participation. Each session lasted approximately 90 min.

Second, in-depth interviews were conducted by the researcher of this study in the professionals’ offices. Three interviews were undertaken with community services social workers and a further three with health and social care workers during the month of March 2019. None of them had participated before in the focus groups. In these interviews, those dimensions that had not been sufficiently explored in the focus groups were addressed. A list of interview questions was not used. Only an opening question was asked: “Tell me about your daily work. How does attending to individuals in situations of vulnerability on a daily basis affect you?” This question facilitated the participants’ discussion. The interviewer took all the necessary notes in a field notebook. The interviews lasted approximately one hour.

In the opinion of the researchers, the two focus groups and the six in-depth interviews were sufficient to achieve data saturation. The possibility of conducting one focus group session in the urban area and the other in a rural municipality was considered in order to identify the differences between community social services in the capital of the province and community social services in a rural setting. Furthermore, both focus groups included not only community social services professionals but also health services professionals in order to have discourses from both types of workers in the two settings studied. Once the focus group sessions were completed, in-depth interviews were conducted to investigate the emerging issues in the focus groups in order to obtain additional data.

### 2.4. Analysis

Giorgi’s method of analysis [50], which involves creating a series of categories and subcategories, was used to analyze the information from both the in-depth interviews and the focus groups. This procedure was carried out in several phases. The first one was an in-depth reading of all the discourses, which had already been transcribed verbatim. The second phase involved a second reading and the division of the data into parts. The basis of the division into parts is meaning discrimination, which presupposes the prior assumption of a disciplinary perspective (social work, in this case). These meaning discriminations constitute parts known as meaning units. The meaning units were examined, tested, and redefined so that the disciplinary value of each unit could be more explicit. These meaning units were then grouped into broader categories according to their shared characteristics and the disciplinary value. In the last phase, the contents of each of the categories were interpreted and analyzed based on the phenomenon or experience lived.

The theoretical–methodological approach was adequate to achieve the objectives of the study. The data obtained were relevant in the context and in other contexts, when compared to the literature.

As for the validity of the results of the analysis, contrast through triangulation was used to control for potential biases resulting from the heterogeneity of the data and the informants’ different points of view. To make a contrast between the differences and similarities conveyed in the discourses, the techniques of focus groups and in-depth interviews were used. In terms of triangulation between subjects, informants were selected from different settings and fields of work to diversify the information present in the discourses regarding the participants’ work experiences in these services. Two researchers began the analysis after the first interview in order to constantly verify that it was in line with the study’s objectives and in order to be prepared in case any change in the research design was needed (it was not). The main categories that researchers identified in the analysis were shared with the participants (by email) to confirm the discourses. In the participants’ discourses where contradictory information was detected, this moment was used to clarify it. The analysis was shared with the rest of the team to ratify the categories. At the same time, an external researcher (with expertise in the subject) validated the analysis.

Reflexivity and a self-critical attitude were maintained throughout the process by all the researchers. To avoid influencing data collection, sample recruitment, and location, the researchers only knew the topic in a superficial manner (as health professionals) and it was not their usual work/subject matter of research.

Atlas.ti 8.0 software (Scientific Software Development GmbH, Berlin, Germany) was used to analyze the discourses.

### 2.5. Ethical Aspects

This research obtained all the necessary authorization from the corresponding Research and Ethics Committee of the University of Almería, Spain (EFM-11/19). Previously, participants had been informed verbally and in writing of the purpose of the study, and their informed consent had been obtained in writing in a dedicated document. The confidentiality and anonymity of participants were preserved throughout this research, in compliance with the bioethical principles of the Declaration of Helsinki [51]. The data from the discourses were safeguarded and protected in accordance with the Spanish regulations in force regarding the official protection of personal data, i.e., the Spanish Organic Law 3/2018, of the 5th of December, on Personal Data Protection and Guarantee of Digital Rights [52].

## 3. Results

The study population comprised 20 professionals: 11 professionals working in community social services and 9 professionals working in the Andalusian Health Service, with a mean age of 46.35 (SD = 7.36) years and with a mean work experience of 24.16 (SD = 7.87) years. Table 1 shows a summary of the sociodemographic characteristics of the sample of professionals who participated in this research.

An analysis of the discourses was performed with the information gathered from the focus groups (FGs) and from the in-depth interviews (IDIs). Three categories with nine subcategories emerged from this analysis. All categories and subcategories were encompassed by a broader category relating to the social workers’ experience (Table 2).

### 3.1. Working with Vulnerable Groups

In day-to-day practice, health and social care workers serve users with very different demands. The characteristics of the population visited by social services are highly varied. Certain settings and realities experienced by users are perceived by social workers as generators of further personal suffering or dismay. Moreover, the traditional social intervention model adopted by social services causes chronic frustration and professional burnout. The main element identified by the informants as a generator of further suffering in the person of the social worker was the intervention work carried out with vulnerable groups.

#### 3.1.1. Minors and the Elderly

Two of the vulnerable groups that had an impact on participants were minors and the elderly, as they are fragile and innocent groups.

*You can’t avoid being touched by the toughest situations, such as those involving minors or the elderly [who are] on their own…* (IDIs, P3).

*…then, well, that… what I always say when there are cases and cases, when you see the despair of a daughter because her mother is ill and the resources she needs do not arrive… There are cases that have an impact, and that no matter how professional you are, you can’t help it, of course not! Because I’m also a person…* (FGs1, P11).

*My weak point, so to speak, is the elderly, especially those who are alone… Many needs arise, and sometimes they cannot be met, and I cannot help but take work home with me…* (FGs2, P17).

*There are users who inevitably impact your situation, or groups such as minors who are still fragile and innocent… And, you find cases where these minors have a rather difficult context and that touches your heart…* (IDIs, P5).

#### 3.1.2. People with Serious Mental Problems

Another group mentioned by participants was people with serious mental problems. The fact of thinking that nobody understands them, that nobody believes them, that they are in danger, that they feel threatened, generates a lot of suffering.

*…above all, patients with serious mental disorders, when they’re having delusions and hallucinations… and it’s so upsetting to think that nobody understands them, that nobody believes them, that they are in danger, that they feel threatened, and that causes me a lot of suffering, and to that, we must add the social aspect, when they see that the life project they had just like everybody else, their dreams… all is shattered…* (IDIs, P5).

#### 3.1.3. People with Scarce or a Lack of Economic Resources

There are also situations of poverty that become permanent for some people and that professionals attend to repeatedly. People with scarce or a lack of economic resources find themselves in very complex situations, and figuring out a way out of these situations has become almost impossible for them, so they visit social workers in a state of desperation, seeking help.

*…these are people who come to my office in great distress because they don’t have the most basic things to eat. I mean, they can’t even pay for water [bills]; they can’t afford the most basic items. Besides, these are chronic situations; they no longer know how to get out of that labyrinth* (IDIs, P3).

*There are families with real hardships; they do not even have a snack for their children to eat at school, and they’ve had it for a long time, and that distresses them so much that they come looking for you again and again…* (FGs1, P9).

*We have many users who are poor, but really poor; they do not even have the most basic needs covered…* (IDIs, P4).

*The fight against poverty that leads to social exclusion must be prioritized. We have seen families with children, families who have suffered evictions, people who have reached a situation of poverty without the possibility of any intervention, and who are constantly visiting you out of desperation…* (FGs2, P20).

### 3.2. Emotions Emerging from Working with Vulnerable Groups

Daily contact with situations of suffering can generate a variety of feelings and emotions among health and social care workers. The need arising in professionals to help users who find themselves in a complex situation is evident. However, sometimes social workers encounter a different reality, and aid does not arrive as expected, thus generating various emotions in them.

The most common emotions expressed by professionals, when facing users’ serious situations or when the outcome is not as expected, included anger, sadness, fear, and concern.

#### 3.2.1. Anger

In relation to anger, participants commented on how seeing injustice, because things are not done as they should be, for example, made them feel helpless, and that helplessness generates anger.

*…and many times, you feel angry and helpless; of course you feel that [way], and whoever says they don’t is lying, because we [usually] see very tough situations…* (FGs2, P14).

*…sometimes I get angry; other times I get sad… It’s a constant state of alertness. That’s my natural state, [and it has been] for some time now* (FGs1, P12).

*When you see injustice, I feel tremendous anger; it makes me very angry that things are not done as they should… or that the response to a user is not what he needs* (IDIs, P6).

*There are days when you get very angry or upset about certain situations that we have to deal with…* (IDIs, P2).

#### 3.2.2. Sadness

The fact of witnessing difficult times that other people go through, or the despair they experience, generates sadness in social workers.

*…I feel like… how can I put it into words?… in a pyramid of dissatisfaction, in the sense that, you know, although you do everything you can, [you see] the outcome in the very long term, and then, in the meantime, you see those people here every day…* (IDIs, P2).

*…other times I have feelings of sadness…* (FGs2, P19).

*It is inevitable to feel sad on many occasions, when users are desperate and the answers do not come…* (IDIs, P1).

*Sometimes there are cases, people, who are in a difficult moment of their lives, and when they share their story with you, you feel a lot of sadness, although you cannot transmit it to them, but inside, you get sad…* (FSs2, P18).

#### 3.2.3. Fear

In some cases, some participants even mentioned the word “fear,” although they did not delve into that emotion.

*…powerlessness, frustration, or even fear…* (FGs2, P17).

*…and so, it scares me…* (FGs2, P15)

*There are situations where you feel fear…* (IDIs, P3)

#### 3.2.4. Concern

Social workers disclosed that there are situations they cannot possibly stop thinking about, such as people who find themselves in very serious circumstances. These are extraordinary cases that social workers keep thinking about, even after their working hours, because, according to their accounts, some issues inevitably haunt them owing to their significance or complexity. The emotion that emerged related to this was concern about the problems of the users.

*…but there are times and situations, quite exceptional ones, that I can’t help remembering; you definitely remember them… There are situations that I still do take home with me, although I’ve been [working this job] for many years, and you have to learn not to take [these situations] with you. Two, three…? or more [of these situations] a year, at least* (FGs1, P13).

*I guess situations stop shocking you with the passage of time, or you see it differently… But that does not mean that there are no cases that do not affect me, or that I [don’t] take them home, flitting around in my head… and you mull over them, or even after some time, you would remember that nothing could be done, you see that family member on the street and you remember. There are always situations that affect you…I don’t know…* (IDIs, P1).

*…I take problems home with me because [first] we’re people and then we’re professionals, and you’re there [trying to] figure out how to solve that situation…* (FGs 1, P12).

Social workers identify the need and the urgency of some situations for some vulnerable groups. Not being able to respond adequately, because sometimes resolving a demand takes time, generates discomfort and concern among professionals.

*…although you do everything you can, [you see] the outcome in the very long term…* (IDIs, P2).

*…when urgent cases arise and you can’t resolve them with the same urgency…you go home thinking “[hopefully] nothing [bad] happens by tomorrow,” and, well, you don’t even know if that’ll be resolved the next day* (FGs2, P20).

*…and when the user leaves, I think, what if by the time it’s resolved it’s too late…? And I know it’s not my fault, but I’m the one who’s facing the music…* (FGs 2, P18).

*…because I have cases [i.e., users] that you attend to and you tell them, “Come back tomorrow to finish this,” or that they have to wait for such-and-such…* (FGs2, P18).

### 3.3. Need for Spaces for Self-Care

Health and social care workers recognized that working with vulnerable groups causes them many negative emotions, as we have previously described. They said that they feel the need to express and share those feelings.

#### 3.3.1. Mutual Support

They end up developing more informal strategies, such as mutual support. Sharing complex cases and learning from the experience of other professionals and their way of dealing with different situations are two of the most valued strategies according to the discourses. Peer support, talking, and discussions of experiences are highlighted and widely accepted by all social workers. They agree that talking to peers and team support are essential to address certain cases or avoid being affected by them in one way or another. Sharing experiences with peers who have undergone similar situations is described as an informal therapy that social workers use to cope with daily work.

*…we support each other here and help each other quite a lot; the director is always there… and that makes a big difference. Maybe peer support is a useful tool to deal with the most vulnerable situations, or so that the most complex cases don’t affect you that much* (IDIs, P6).

*…[having a] good relationship with my peers always helps; for me that’s my therapy* (FGs2, P15).

*Sharing experiences with colleagues is a mechanism that comes in handy so as to manage all these situations of frustration or in order to consider what [other] alternatives may exist, in addition to the ones you already know* (IDIs, P4).

*…we are like a kind of group therapy, and [I have] wonderful colleagues; we support each other, really, at least in my experience* (FGs 2, P13).

*If there is a case that worries me, she always asks me the first one, always, and I feel very supported. Also, in this office, [which] is shared with another colleague, if you arrive from a bad day, especially tired from so many kilometers, we can share how the day has gone and we can let off steam between us* (IDIs, P4).

*The truth is that relationships with colleagues are very good, and you find support, and of course that matters; just talking and venting our feelings already help* (IDIs, P1).

*…I have colleagues who may have [many] more years of experience or who have already undergone a similar case and similar experiences. It’s always good that they give you, like, their insight* (FGs1, P12).

#### 3.3.2. Formal Spaces to Work Emotions

Professionals talk about the benefits of peer support and report that it exists and is real. However, they also emphatically express the need for training and support in formal settings to learn how to deal with certain cases or not to bring those situations to their personal lives. They recognize the need to develop other types of skills to help them manage their own suffering and dissatisfaction in structured spaces dedicated to training, and emotional support. Receiving training and support in formal spaces can be positive. Working with emotions could favor their work and their professional and personal quality of life. Informants demand that social workers be cared for so that their work, which is in contact with suffering, is sustainable, without becoming exhausted or burned out.

*…from the upper management, they have to think about the professionals; we lack the tools to face the current situations we are experiencing…* (FGs2, P15).

*Yeah, why not? Spaces to work on emotional education or other types of therapies and teachings so as to care for professionals, [and] formal spaces to talk to peers. These can be things that greatly facilitate and favor the work of social workers and their professional and personal quality of life. Psychologists and others to be within our reach… All professionals need their own space, and I think that in the long run it may prove useful, so why not?* (IDIs, P1).

*I believe that emotional education and self-care must be present… emotional education must always be present, on a professional and [a] personal level. Spaces where we can formally work with our colleagues…* (FGs2, P12).

*…maybe it would be good to have a structured space for the self-care of professionals* (FGs1, P13).

*A space to take care of oneself would be great, spending time with each other while learning to work with emotions* (IDIs, P5).

## 4. Discussion

Social workers constantly provide care to people in vulnerable situations with complex demands. This scenario causes suffering in professionals.

In the literature consulted, social workers are portrayed as resource providers [17], that is, as mere intermediaries between the group of individuals with needs and the resources that the state decides to allocate. As a result, neither deadlines nor requirements depend on social workers themselves [53]. The professionals in our study and those in previous studies [54] agree that the bureaucratic processes faced by users represent obstacles for social workers as well, who have the feeling of not stepping in on time.

Social services users are very varied. The needs of each individual are different, and not all of these needs are equally demanding [9]. Among the plethora of cases, some groups are more vulnerable than others [38]. In concordance with our research, other studies have also shown that minors and the elderly are considered to be among the most vulnerable populations [24]. In addition, health and social care workers point out that individuals with mental illnesses [22] and individuals who are in a chronic situation from which they cannot get out are groups at greater risk [55,56].

As shown in this research, the emotions generated in professionals who are in constant contact with situations of suffering with a high emotional impact, such as the aforementioned, originate deep frustration, anger, and dissatisfaction, as well as sadness, fear, and concern. This is in consonance with the literature consulted, which also reports the great effect that working daily with the intense suffering of users has on social workers, along with a potentially poor response [26,27,57,58]. In fact, some studies conceptualize emotion as both a potential resource and a risk for social workers’ professional judgment and practice [59].

The social workers in our study considered that the most complex interventions with vulnerable people inevitably make it difficult for them to switch off from work. This aspect is consistent with other studies in which social workers had difficulties when switching off from work after attending to groups with complicated needs [55,60]. With regard to the reported resources for self-care, mutual support is virtually the only helping tool available to these professionals. In previous studies, professionals referred to social support as a key element [13,27,43] but did not specifically mention mutual peer support. Social workers ask for training and support in the face of complex social interventions, as proposed by different research studies that underline the importance of taking care, preparing, training, and supporting social workers in this regard [61,62]. Therefore, more attention should be paid to working with the emotions of social workers who are exposed to tense and threatening situations [63,64]. In this way, for instance, reflexivity strategies in order to build and rebuild emotions in social workers could be useful [65]. Receiving training and support in formal spaces, as social workers described in this study, could be positive for their work and their professional and personal quality of life.

As for the limitations of this study, we considered the possibility that the researchers’ personal positions on the matter may have introduced bias into the results. To control for bias, reflexivity and a self-critical attitude were maintained throughout the process by all the researchers. To avoid influencing data collection, sample recruitment, and location, the researchers only knew the topic in a superficial manner (as health professionals) and it was not their usual work/subject matter of research. We relied on the ultimate motivation for our work, which is to acquire knowledge to improve, rather than to demonstrate. Nevertheless, we have set out to conduct a release exercise to clarify our own assumptions and put them into perspective when designing our research.

Regarding future lines of research, a study with the methods combined regarding the quality of professional life of social workers should be conducted in order to identify related factors and to assess the levels of compassion fatigue. In addition, research should be undertaken on the concept of compassion among social workers and its relationship to suffering. Finally, interventions should be carried out with social work professionals to develop compassion as a protective element against compassion fatigue.

## 5. Conclusions

Social workers experience high levels of emotional discomfort when carrying out their work, which is exacerbated when the populations they attend to are particularly vulnerable groups, such as children, the elderly, individuals with mental health problems, or people with scarce or a lack of economic resources. The traditional hegemonic intervention model that lies within the structure of social services in this context results in all social work efforts revolving around the allocation of available resources, which are generally scarce. All aspects of the individual have been eliminated from the repertoire of interventions according to the comprehensive support approach. In this new model of care, based mainly on providing support to individuals, the professionals themselves are the main tools and resources. In the future, this will further enhance the role of social workers when supporting people experiencing social exclusion, poverty, and marginalization. More attention should be paid to working with the emotions of social workers who are exposed to tense and threatening situations. Peer support, talking, and discussions of experiences are highlighted as relevant to deal with their work. Receiving training and support (in formal spaces) in order to learn how to deal with vulnerable groups could be positive for their work and their professional and personal quality of life.

## Figures and Tables

**Table 1:** Sociodemographic characteristics of participants.

Variables	Focus Group (*N* = 14)	In-Depth Interviews (*N* = 6)
Gender		
Female	12	6
Male	2	-
Marital status		
Married	10	5
Single	3	1
Others	1	-
Work experience (years)		-
10–20	1	1
20–30	3	5
30–40	9	1
>40	1	
Area of work		
Community Services	8	3
Health Services	6	3

**Table 2:** Categories and subcategories emerging from the study.

Categories	Subcategories
Working with vulnerable groups	- Minors and the elderly - People with serious mental problems - People with scarce or a lack of economic resources
Emotions emerging from working with vulnerable groups	- Anger - Sadness - Fear - Concern
Need for spaces for self-care	- Mutual support - Formal spaces to work emotions

## Data Availability

Not applicable.
